# Experimental data validating the optimization of a wireless power transfer prototype employing a novel phase shift measurement system and frequency control

**DOI:** 10.1016/j.dib.2022.108675

**Published:** 2022-10-14

**Authors:** Andrés Martínez, Christian González, Adrián Jaramillo, Dorindo Cárdenas, Alejandro Von Chong

**Affiliations:** a School of Electrical Engineering, Universidad Tecnológica de Panamá, Víctor Levi Sasso Campus, Panama; b CEMCIT–AIP, SNI–SENACYT, School of Electrical Engineering, Universidad Tecnológica de Panamá, Víctor Levi Sasso Campus, Panama; c CEMCIT–AIP, School of Electrical Engineering, Universidad Tecnológica de Panamá, Víctor Levi Sasso Campus, Panama

**Keywords:** Frequency control, Optimization, Phase shift, Resonant, Wireless power transfer, Acquisition

## Abstract

Resonant wireless power transfer (WPT) systems have been evolving and improving their designs over the last few years, looking to efficiently charge electric vehicles, cellphones, and biomedical devices. In this article, we present to the scientific community the data obtained from the optimization of a resonant WPT prototype, operating at different vertical misalignments and load conditions, known to have an impact on the behavior of these type of systems. To maximize the power transferred to the load, we developed a proportional-integral frequency control algorithm that employs the phase-shift between the voltage and current waveforms in the transmitting antenna (resonance indicator) as a setpoint. Data on the performance and control optimization process of the prototype during laboratory tests were acquired using a LabVIEW interface, which was designed to capture information such as the evolution of the frequency, the phase-shift, and the load voltage, from multiple devices (a microcontroller, an oscilloscope, a digital multimeter, and a controllable power supply). The data were organized and presented in tables and graphs using MATLAB. The importance of the dataset relies on the opportunity to utilize the information as a basis for the improvement of the associated electronics by using different transmission topologies, higher speed components, new-generation microcontrollers, and to modelling novel intelligent control algorithms, such as adaptative neuro-fuzzy inference systems.

**Specifications Table**

**Table:** 

Subject	Energy
Specific subject area	Energy Engineering and Power Technology
Type of data	Table Graph Figure CSV files
How the data were acquired	The data were acquired using a LabVIEW interface that collected the information from the outputs of the devices measuring the phase-shift behavior and the voltage response of the wireless power transfer prototype via USB connections. Specifically, the interface stored the frequency and the phase shift computed by the Teensy 4.1 microcontroller after each iteration of the PI controller, and similarly, stored the phase shift pulse width (necessary for calculations) and load voltage, which were captured by the oscilloscope's channels. In addition, the current of the controllable power supply that feeds the system was recorded. Subsequently, the captured data were organized, filtered, and analyzed using MATLAB.The data acquisition system comprises: • Computer with the developed LabVIEW interface. • A four-channel oscilloscope model SIGLENT SDS1104X-E (only two channels were required). • Controllable Power Supply model SIGLENT SPD3303X-E. • Digital Multimeter model SIGLENT SDM3445X. • Teensy 4.1 microcontroller. • USB cables.
Data format	Raw and filtered
Description of data collection	The data presented were collected during multiple laboratory tests, in which the wireless power transfer prototype was operating under different misalignment and load conditions. A P-I frequency control algorithm was implemented to optimize the power transfer in each test. The evolution of the frequency, phase-shift, and load voltage over time was stored with the help of the data acquisition system. Six load conditions were evaluated: 1. 100-ohm resistor 2. 200-ohm resistor 3. 500-ohm resistor 4. 1,000-ohm resistor 5. 1,500-ohm resistor 6. 2,000-ohm resistor Furthermore, three vertical misalignments between the transmitting and receiving coils of the prototype were assessed for each load: 1. z = 0 mm 2. z = 4 mm 3. z = 8 mm
Data source location	• Institution: Universidad Tecnológica de Panamá • City/Town/Region: Panama, Panama • Country: Panama
Data accessibility	Repository name: Mendeley Data identification number: doi:10.17632/74ptpjsd5z.3 Direct URL to data: https://data.mendeley.com/datasets/74ptpjsd5z/3
Related research article	A. Martínez, C. González, A. Jaramillo, D. Cárdenas & A. Von Chong, Low-cost, microcontroller-based phase shift measurement system for a wireless power transfer prototype, HardwareX, 11, e00311. https://doi.org/10.1016/j.ohx.2022.e00311.

## Value of the Data

• The reported data provides the electrical parameters used to assess the performance of a series-series compensated WPT circuit, which can be contrasted against other WPT compensation topologies.
• Data at different load conditions and vertical misalignments were collected under identical conditions. This allows to further study and evaluate the mathematical modelling for the electromagnetic phenomena involved.
• The data can be used as a basis for the improvement of the system, such as switching to higher-speed electronic components or using newer-generation microcontrollers.
• A novel method for the estimation of the phase angle for WPT applications is demonstrated by providing data on the optimization of the power transferred via a PI algorithm. This data could be used to design an alternative robust and adaptive control algorithm.

## 1 Data Description

Wireless power transfer (WPT) is an emerging yet promising technology that improves electrical energy applications and consequently enhances human life. Ever since it was demonstrated that the energy could be efficiently transmitted between two coils separated 2 meters using resonant coupling in 2007 [1], the scientific community have been devising novel topologies and prototypes to ameliorate the performance of resonant WPT systems. Nowadays, we find this technology in commercial products such as electric toothbrushes, power mats for cellphones, biomedical devices, and even chargers for electric vehicles [2]. Furthermore, WPT technology plays a crucial role in solving the issues of energy crisis and environmental pollution [3].

The data presented in this article are the validation results of a wireless power transfer (WPT) system presented in [4]. In WPT systems, maximum power transfer occurs at the resonance frequency (which is the desired optimal operating condition). The value of this frequency varies when the coils are misaligned, which is a common problem in real-life applications. To increase the efficiency of the system, a proper tracking of the voltage-current phase angle is needed. In [4], a phase angle detection technique uses an XOR gate to generate a pulse (HIGH) when the voltage and current signals have different signs (either positive or negative) and (LOW) when both possess the same sign. The width of this pulse is a proxy to the phase angle, and the proposed system uses a PI controller to modify the frequency seeking to narrow the pulse's width.

The datasets presented in this article can be accessed through the online data repository found in [5]. The data were acquired with a custom data acquisition software available at: WPTDevice2.0>LABVIEW_INTERFACE>AcquisitionSoftware.vi in [4]. This section provides details about the information contained in the files, as well as presents graphs generated from the analysis of the data.

## 2 Folder Name: ‘SourcetxtFiles’

The SourcetxtFiles folder contains the raw data obtained by the acquisition software, and holds all the tests performed, in a text (.txt) format.

Frequency sweep and power transfer optimization tests were performed via PI controller for three vertical misalignments named z0, z1, and z2 (0 mm, 4 mm, and 8 mm correspondingly), with six different loads: 100-ohm, 200-ohm, 500-ohm, 1,000-ohm, 1,500-ohm, and 2,000-ohm resistors. This yields a total of 36 tests.

Each subfolder z0, z1, and z2 has the files listed below. Note that [X] represents the values of the different loads in ohms.

• *DataExtract.m*: MATLAB script required to perform the data import, data filtering, and Excel (.xlsx) file generation from the source text (.txt) files.
• *WPTTeensySweep [X].txt*: data acquired by the acquisition software during a frequency sweep ranging from 50 kHz to 90 kHz.
• *WPTTeensyPID [X].txt*: data acquired by the acquisition software for a power transfer optimization via a PI controller.

## 3 File Name: “[Data descriptor] [Load condition] [Vertical misalignment].xlsx”

After executing the *DataExtract.m* script with MATLAB for the beforementioned source text (.txt) files, twelve Excel (.xlsx) are generated, which have the following syntax:

The [*Data descriptor*] prefix can have the following values:

• *DMMLoadVoltage*: Load's RMS voltage measured with the SIGLENT SDM3445X digital multimeter.
• *MovingMedianPhaseAngle*: Data on the median angle computed by the Teensy 4.1 microcontroller, filtered with a moving median filter of size 6.
• *NextPWMsent*: Data on the frequency of the PWM signal sent by the Teensy 4.1 microcontroller.
• *ONdutycycle*: Data on the ON duty cycle of the XOR's gate output (*i.e.,* the phase-shift pulse width) measured by the SIGLENT SDS1104X-E oscilloscope.
• *OscilloscopeLoadVoltageSignal*: Data on the voltage signal at the load, measured by the SIGLENT SDS1104X-E oscilloscope. Data contains 7000 samples obtained continuously during 35 microseconds.
• *OscilloscopePhasePulseSignal*: Data on the XOR's logic gate output (*i.e.,* the phase-shift pulse width), measured by the SIGLENT SDS1104X-E oscilloscope. Data contains 7000 samples obtained continuously during 35 microseconds.
• *OscilloscopeRMSLoadVoltage*: Data on the computed on the load's root mean square voltage. Data were generated with the math function present in the SIGLENT SDS1104X-E oscilloscope.
• *PhaseAngleRaw*: Unfiltered phase angle computed by the Teensy 4.1 microcontroller.
• *PWMmeasured*: Data on the measured PWM output via the rising edges count.
• *RawData.mat*: The raw and filtered data generated from the .txt source files, as a MATLAB file.
• *toff*: The XOR's logic gate output (*i.e.,* the phase-shift pulse width), off time, computed by the Teensy 4.1 microcontroller.
• *VoltageSourceCurrentOutCh1*: Current output measured by the channel 1 of the voltage source SIGLENT SPD3303X-E.
• *VoltageSourceCurrentOutCh2*: Current output measured by the channel 2 of the voltage source SIGLENT SPD3303X-E.
• *VoltageSourceVoltageOutCh1*: Voltage output measured by the channel 1 of the voltage source SIGLENT SPD3303X-E.
• *VoltageSourceVoltageOutCh2*: Voltage output measured by the channel 2 of the voltage source SIGLENT SPD3303X-E.

The [*Load condition*] infix can have the following values:

• 100Ohm: Test performed with a 100-Ohm resistor.
• 200Ohm: Test performed with a 200-Ohm resistor.
• 500Ohm: Test performed with a 500-Ohm resistor.
• 1000Ohm: Test performed with a 1000-Ohm resistor.
• 1500Ohm: Test performed with a 1500-Ohm resistor.
• 2000Ohm: Test performed with a 2000-Ohm resistor.

The [Vertical misalignment] suffix can have the following values:

• 0mm: Test performed with a vertical misalignment of 0 millimeters.
• 4mm: Test performed with a vertical misalignment of 4 millimeters.
• 8mm: Test performed with a vertical misalignment of 8 millimeters.

**Fig. 1:**
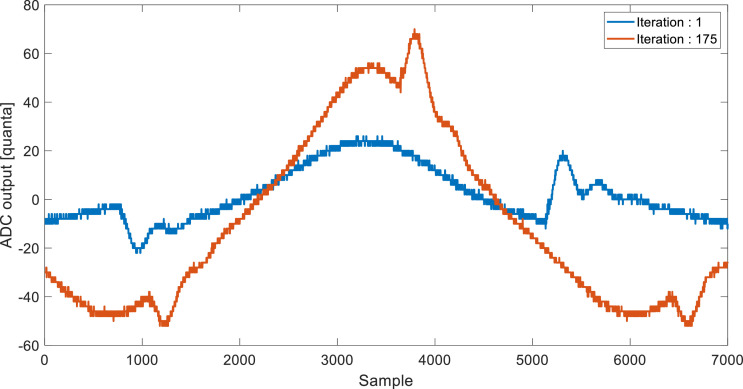
Load voltage signal during a phase angle optimization routine via a PI controller for a resistive load of 500-ohm and a vertical misalignment of 4 mm.

Fig. 1 shows the behavior of the load voltage for iteration 1 (blue) and iteration 175 (orange) of the phase angle optimization algorithm. For this experiment, the coils were separated 4 mm and a 500-ohm resistor was used. Signals were obtained with a SIGLENT SDS1104X-E oscilloscope.

**Fig. 2:**
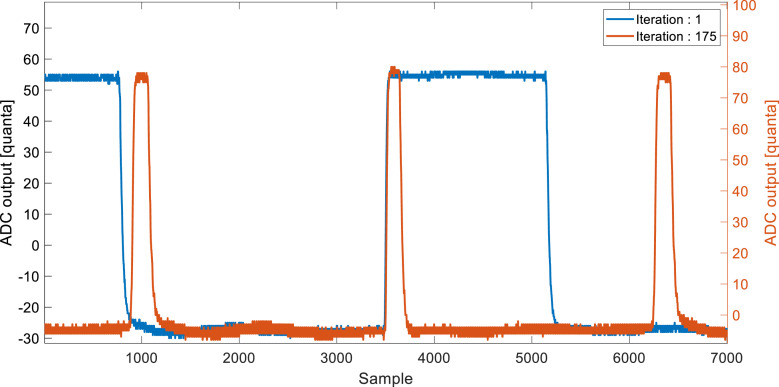
XOR's logic gate output for a PI optimization routine for a resistive load of 500-ohm and a vertical misalignment of 4 mm.

Fig. 2 contrasts two phase angle pulse widths. The blue signal represents a non-optimized operation frequency at the beginning of the test (iteration 1), whereas the orange signal is the optimized signal at iteration 175 of the PI loop. Both signals were obtained with a SIGLENT SDS1104X-E oscilloscope with the probe placed at the exclusive OR gate's (XOR) output.

**Fig. 3:**
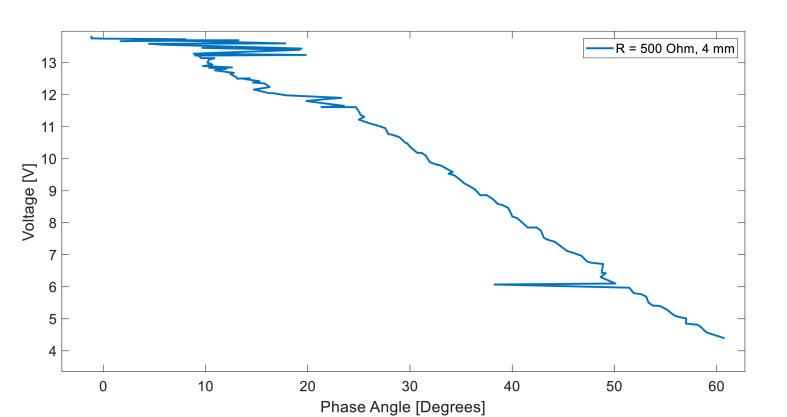
Load voltage vs. phase angle for a PI optimization routine for a resistive load of 500-ohm and a vertical misalignment of 4 mm.

**Fig. 4:**
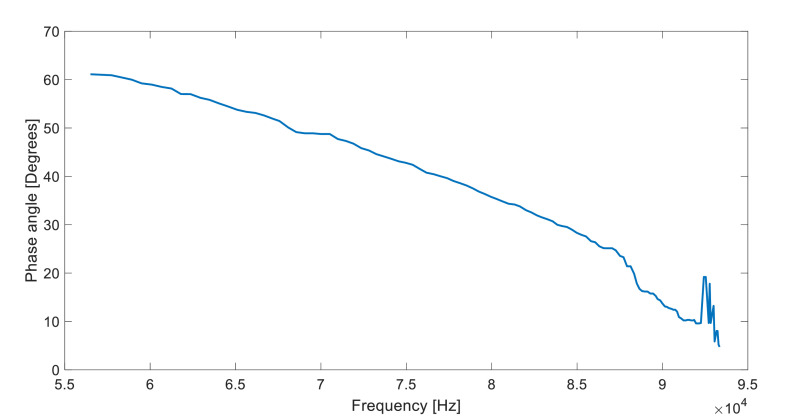
Phase angle vs frequency for a PI optimization routine using a 500-ohm resistor and a vertical misalignment of 4 mm.

Fig. 3 depicts the evolution of the load voltage in a 500-ohm resistor while optimizing the phase angle (iteration 175). A vertical separation of 4 mm was introduced between the transmitting and receiving coils. For this test, Fig. 4 shows the minimization of the phase angle as a function of the input frequency (dependent vs. independent variables).

**Fig. 5:**
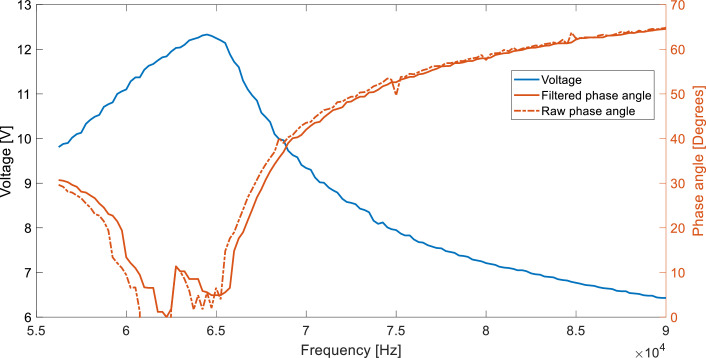
Frequency sweep for a 100-ohm resistive load, with a vertical misalignment of 0 mm.

Fig. 5 exhibits the voltage and phase angle (raw and filtered) response in a 100-ohm load during a frequency sweep test. For this sweep, no vertical misalignment was implemented.

## 4 Experimental Design, Materials and Methods

**Fig. 6:**
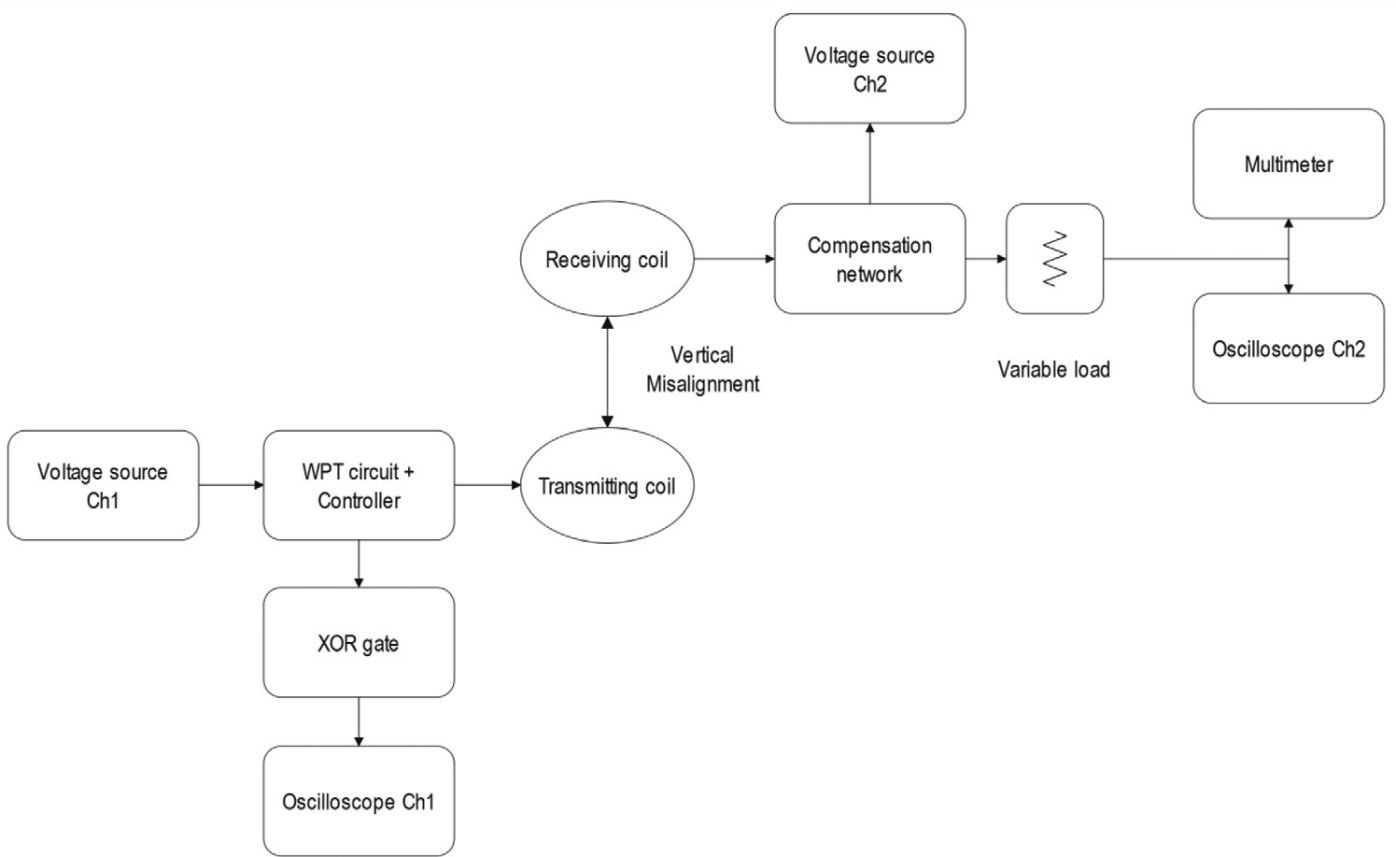
Block diagram of the data acquisition setup for the WPT optimization tests.

**Fig. 7:**
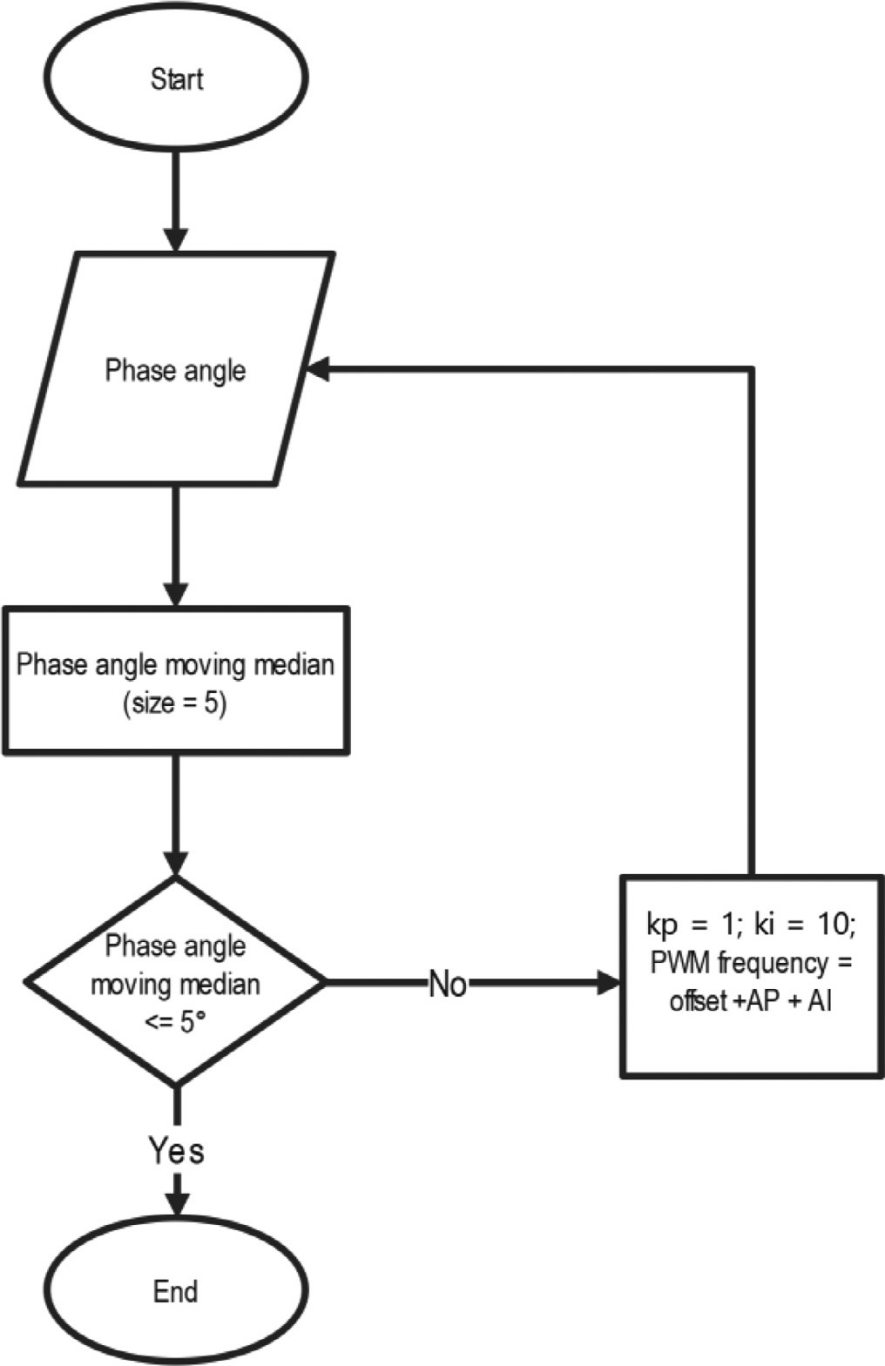
Flow chart of the iterative process of the controller for the WPT optimization tests.

The electronic circuit for the wireless power transfer prototype, along with the data acquisition software can be found in [4]. Considering this, this section will further describe the embedded algorithm and the data collection process. The data acquisition setup is presented in Fig. 6. Firstly, the vertical misalignment (independent variable) is set to one of the z0, z1 or z2 value. Subsequently, the transmitting coil is powered with a PWM signal, and the phase shift measurement system starts to read the phase angle (dependent variable) between voltage and current waveforms through the output of a XOR gate. These measurements are captured by Ch1 of a SIGLENT SDS1104X-E oscilloscope and exported to a LabVIEW interface. The microcontroller (Teensy 4.1) then executes an embedded algorithm to modify the PWM signal frequency (independent variable, control variable) thus modifying the phase angle. The algorithm is thoroughly explained in the next subsection. While operating the primary side, the receiving coil and the compensation network feed the resistive load. The voltage and frequency (dependent variables) of this load are captured by a SIGLENT SDM3445X digital multimeter and the Ch2 of the oscilloscope. A flowchart of the iterative process is shown in Fig. 7.

## 5 Embedded Algorithm

For the validation of the proposed method, a PI (proportional-integral) algorithm was embedded in a Teensy 4.1 microcontroller to adjust the operating frequency (independent variable), seeking to minimize the phase angle (dependent variable). The output of this PI algorithm was sent to the transmitting coil via a digital output pin of the microcontroller as a PWM signal using the analogWriteFrequency() function. For all the tests, the *kp* and *ki* values were set to a constant value of 1 and 10 correspondingly. The PI algorithm's output was calculated as *freqcalc*=*f_init+AP+AI*, where *freqcalc* is the updated frequency value of the generated signal, *f_init* is a constant value of 50,000 (data becomes relevant for values above 50 kHz), *AP* is the error times *kp* (where *error*=*angle*, since the setpoint is always zero), and *AI* is the cumulative sum of *error* times *ki*. The phase angle is calculated as *angle* = 360.0*(0.5-(flanks*toffavg/1000000). *flanks* is the measured frequency output at the PWM pin and *toffavg* is the off-time average (the duty cycle's complement) at the XOR gate's output signal. *toffavg* is measured using the Arduino's *pulsein()* function. The algorithm discards the off-time values that are greater than the XOR output signal's period.

## 6 Data Acquisition

The data acquisition was done with the WPT circuit energized, the Teensy 4.1 microcontroller, and all the measuring instruments (*i.e.*, the SIGLENT SDM3445X digital multimeter, the SIGLENT SDS1104X-E oscilloscope, and the SIGLENT SPD3303X-E power supply) turned on. All the measuring instruments were plugged via USB to the computer running the acquisition software. Handshaking between the microcontroller and the measuring instruments was controlled by the LabVIEW interface, available at [4]. With this interface, the synchronization of the data from the Teensy is activated and stopped when the operator decides to. Each iteration of the PI algorithm was done with a 1 second delay, to allow sufficient time for the instrument's acquisition time, data communication, and data storage on the PC.

The data is obtained in the following way:

• Oscilloscope's channel 1: Receives the voltage pulses from the output of the XOR gate which represent the phase shift width.
• Oscilloscope's channel 2: The voltage from the load (resistor) located at the secondary side module.
• Controllable power supply: Data on the delivered currents and voltages are registered. Voltage measurements are useful to make sure the WPT circuit is not on short circuit.
• Teensy 4.1: Receives the signals from the output of the XOR gate at the phase shift measurement module.

This data is then stored and saved as a .txt file if the “Data storage” button is activated.

## 7 Data Filtering

Following the acquisition process, all the data were moved from the text files generated by the LabVIEW interface to MATLAB. Subsequently, the data were imported, organized, and filtered with a MATLAB script (MATLAB version R2020b). Filtering was necessary to remove the outliers introduced by the data losses during serial communication. To remove the outliers, the MATLAB script first determined the instances in which a value of zero was registered, changed it to NaN (not a number), and the fillmissing() function was used to interpolate the NaN values via linear interpolation. The data filtering MATLAB script is included in the repository in:

Experimental DataWPT>SourcetxtFiles>z0>DataExtract.m.

## Ethics Statements

No ethics fields involved.

## CRediT authorship contribution statement

**Andrés Martínez:** Conceptualization, Methodology, Software, Validation, Investigation, Writing – original draft, Writing – review & editing, Visualization. **Christian González:** Conceptualization, Methodology, Software, Validation, Investigation, Writing – original draft, Writing – review & editing, Visualization. **Adrián Jaramillo:** Methodology, Software, Resources, Validation, Investigation. **Dorindo Cárdenas:** Conceptualization, Methodology, Supervision, Writing – review & editing. **Alejandro Von Chong:** Conceptualization, Methodology, Supervision, Investigation, Software, Validation, Resources, Writing – review & editing, Project administration.

## Declaration of Competing Interest

The authors declare that they have no known competing financial interests or personal relationships that could have appeared to influence the work reported in this paper.

## Data Availability

Experimental data validating the optimization of a wireless power transfer prototype employing a novel phase shift measurement system and frequency control (Reference data) (Mendeley Data).
